# Exploring skin adverse events and mechanisms of apalutamide using data mining algorithms and network pharmacology

**DOI:** 10.3389/fphar.2025.1517874

**Published:** 2025-02-13

**Authors:** Yaqing Chen, Longzhuan Huang, Wenwei Li, Hangye Gu, Yong Chen

**Affiliations:** Key Specialty of Clinical Pharmacy, The First Affiliated Hospital of Guangdong Pharmaceutical University, Guangzhou, Guangdong, China

**Keywords:** apalutamide, Stephen Johnson Syndrome/toxic epidermal necrolysis, JAK, FAERS, pharmacovigilance

## Abstract

**Background:**

Skin adverse events of apalutamide pose a major challenge to its clinical use, particularly the severe and difficult to identify toxic epidermal necrolysis. For the purpose of providing the basis for the clinical monitoring of the administration of apalutamide and further research. This study examined the pathways of apalutamide and Stephen Johnson Syndrome/Toxic Epidermal Necrolysis using network pharmacology and data mining tools to analyze skin adverse events.

**Methods:**

Using the Information Component method and the Reporting Odds Ratio, the relationship between apalutamide and skin adverse events was evaluated. Molecular docking was utilized to explore the potential mechanism of apalutamide and Stephen Johnson Syndrome/toxic epidermal necrolysis.

**Results:**

With a median time to onset of all skin adverse events of 55 days, a total of 21 skin-related adverse events were found. Low body weight and advanced age may be major hazards for skin adverse events with apalutamide. The results showed a substantial association between apalutamide and Stephen Johnson Syndrome/toxic epidermal necrolysis, and the mechanism behind this association may be the binding of apalutamide to JAK1 and JAK2.

**Conclusion:**

Special attention is recommended for skin adverse events when using apalutamide, especially for rapidly progressing and severe adverse events. To confirm the connection between the triad of Janus kinase, apalutamide, and skin adverse events, further research is required in the future.

## 1 Introduction

Men are afflicted with prostate cancer globally, which is one of the most common cancers to be diagnosed with. It is also one of the cancers that kill the most people worldwide, with the fifth highest death rate (Sung et al., 2021). The cornerstone treatment of prostate cancer predominantly relies on androgen deprivation therapy, initially exhibiting promising clinical efficacy. Notwithstanding, prostate cancer can engage diverse mechanisms to activate the androgen receptor (AR), resulting in the formation of AR-androgen complexes which can be translocated into cells and modulate gene expression (Dai et al., 2017). This intricate cascade ultimately engenders the recurrence and progression of prostate cancer. Studies have demonstrated that direct AR antagonists have displayed noteworthy efficacy in the treatment of prostate cancer. These AR antagonists work by binding competitively to the AR, thereby blocking the androgen-mediated process and inhibiting the proliferation of prostate cancer cells (Helsen et al., 2014; Kim et al., 2021). However, when the AR overexpressing, first-generation AR antagonists are prone to resistance and manifest a combination of antagonist and agonist effects (Hodgson et al., 2005; Suzuki et al., 2008; Kawata et al., 2010).

Apalutamide, a second-generation selective androgen receptor antagonist, employs a comprehensive mechanism of action to effectively inhibit the activation of the AR signaling pathway. This inhibition leads to a notable deceleration in the progression of prostate cancer (Tran et al., 2009; Rajaram et al., 2020). Apalutamide has received endorsement from multiple guidelines, such as NCCN and CSCO, as a first-line treatment option for metastatic hormone-sensitive prostate cancer, nonmetastatic castration resistant prostate cancer, and metastatic castration resistant prostate cancer.

Despite the widespread utilization of apalutamide in prostate cancer treatment, skin adverse events (AEs) pose a significant challenge to its clinical use (Wang et al., 2024; Yang et al., 2024), especially with the addition of Stevens-Johnson syndrome (SJS) and Toxic Epidermal Necrolysis (TEN) in the 2022 specification. SJS and TEN are within the same spectrum of disease, but the area of epidermal exfoliation differs between them, with TEN occurring when the area of exfoliation is >30% of the body surface area. SJS/TEN is a rare, drug-induced, rapidly progressing disease (Schwartz et al., 2013; Gibson et al., 2024). Therefore, a retrospective analysis was conducted to examine the incidence of skin adverse events associated with apalutamide in comparison to other FDA-approved drugs. The analysis was performed using the US Food and Drug Administration Adverse Event Reporting System (FAERS), a pharmacovigilance database. Additionally, network pharmacology was employed to investigate the potential association between apalutamide and SJS/TEN, with a view to informing clinical medication monitoring and ongoing research.

## 2 Materials and methods

### 2.1 Data source

The data utilized in this study were sourced from the FAERS database, an openly accessible repository designed for the submission of adverse events by the general populace. Each quarterly data file within the database encompasses essential documents, including DEMO (which contains patient demographic and administrative information, a single record for each event report), DRUG (which contains drug/biologic information for as many medications as were reported for the event), REAC (which contains all AEs), INDI (which contains indications for use (diagnoses) for the reported drugs), THER (which contains drug therapy start dates and end dates for the reported drugs), OUTC (which contains patient outcomes for the event), RPSR (which contains report sources for the event), data usage instructions, and deletion reports.

**FIGURE 1:**
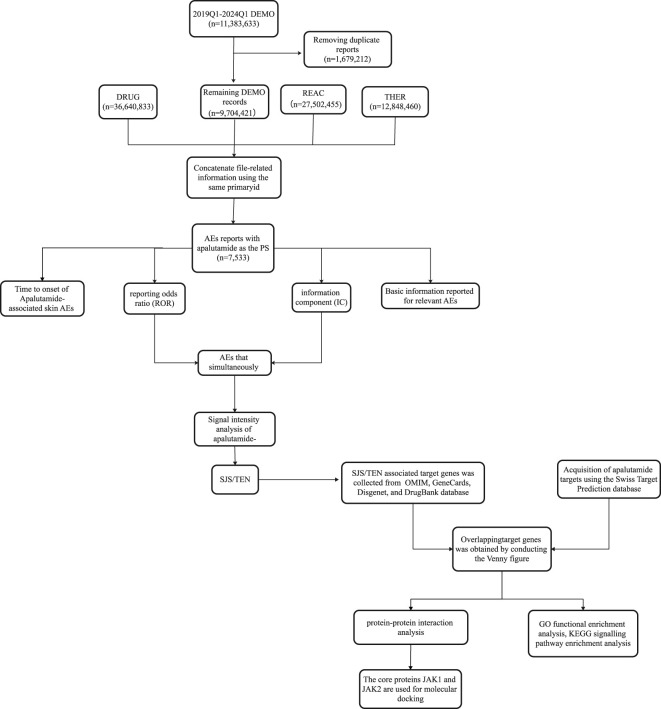
Flow diagram of this study.

Apalutamide was initially launched in the US in February 2018, so data from its sixth anniversary was included in this study for analysis (Q1 2018 to Q1 2024). Considering the recommendations of FAERS in the Q1 2004 and Q1 2019 guidance documents, we de-emphasized the data in the demo file with the de-emphasis details: Retain the report with the highest FDA_DT value among those with the same caseid. If the caseid and FDA_DT are identical, retain the report with the greatest primaryid, and use the same primaryid to map and match the report data (Figure 1).

The generic and proprietary designations (apalutamide, Erleada) were employed as search criteria for the data extraction process. Consideration was solely given to reports in which apalutamide was listed as the “primary suspect”. Adverse Events (AEs) reports within the FAERS database are encoded using Preferred Terms (PTs) as prescribed by the Medical Dictionary for Regulatory Activities (MedDRA). While different PTs are organized into varied System Organ Classes (SOCs), a principal SOC exists, reflecting the feature known as multiaxiality. The inclusion criteria were determined by the SOC equating to “Skin and subcutaneous tissue disorders” and the primary SOC being marked as “Yes”.

### 2.2 Datamining

In an effort to contrast the susceptibility to skin adverse events among individuals treated with apalutamide versus instances reported from alternate pharmacological interventions within the database, a disproportionality study was instituted employing reporting odds ratio (ROR) in conjunction with information component (IC). To eliminate the generation of false positive signals, this study included the analysis of the signal generation conditions following the above two methods, the principles of the two methods and the signal generation conditions are shown in Tables 1, 2. A signal is elicited under the circumstance where both methodological standards are simultaneously satisfied, thus pointing to statistically special associations (Huang et al., 2014; Caster et al., 2020).

**TABLE 1:** Disproportionality method four-cell table.

Events	Number of target ADE reports	Number of other ADEs reports	Sum
Target drug	a	b	a + b
Other drugs	c	d	c + d
Sum	a + c	b + d	a + b + c + d

**TABLE 2:** Calculation formulas and judgment conditions of the corresponding algorithms.

Methodologies	Formula	Judgment conditions
ROR	$\text{ROR}=\frac{\left(\frac{a}{c}\right)}{\left(\frac{b}{d}\right)}=\frac{ad}{bc}$ $95\%CI=e^{\ln\left(ROR\right)\pm 1.96\sqrt{\left(\frac{1}{a}+\frac{1}{b}+\frac{1}{C}+\frac{1}{d}\right)}}$	a ≥ 3, lower limit of 95% confidence interval for ROR ≥ 1, then one signal is generated
IC	$IC=\log_{2}\frac{a\left(a+b+c+d\right)}{\left(a+b\right)\left(a+c\right)}$	a ≥ 3, IC-2SD > 0, then one signal is generated

Meanwhile, we used the chi-square test to investigate the relationship among different age groups, body groups, and skin adverse events, and we reviewed reports with comprehensive data records to determine the induction time of adverse events associated with skin toxicity. In addition, using the difference between the date of treatment initiation (START_DT in the THER file) and the date of the adverse event (EVENT_DT in the DEMO file), we computed the median and interquartile range of ADE induction times for skin toxicity related ADEs by analyzing reports with complete data records. Using R software version 4.4.1, Weibull distribution analysis was further carried out to model the variation in the occurrence of adverse events across time.

### 2.3 Network pharmacology

In this study, network pharmacology was utilized to explore the potential mechanism of SJS/TEN caused by apalutamide. The molecular structure and target genes of apalutamide were searched from the PubChem database and the Swiss Target Prediction database (Daina et al., 2019; Kim et al., 2023), respectively. Information about SJS/TEN associated target genes was collected from disease databases, including the online Mendelian inheritance in man database (OMIM), GeneCards, Disgenet, and DrugBank database (Stelzer et al., 2016; Piñero et al., 2019; Knox et al., 2024; McKusick-Nathans Institute of Genetic Medicine, 2024). Target genes with relevance scores greater than zero were filtered in the GeneCards database and obtained from other databases using default values, then aggregated and duplicates removed. The overlapping target genes of SJS/TEN and apalutamide were obtained by conducting the Venny figure (Oliveros, 2015). The protein-protein interaction map was constructed to find key proteins, then using the string database, and the species was set to “*Homo sapiens*”, with a confidence score of 0.4, and removing 2 proteins that were not connected to the network graph, other parameters remain at their default settings (Szklarczyk et al., 2023). Gene Ontology (GO) and Kyoto Encyclopedia of Genes and Genomes (KEGG) analyses were performed on the overlapping targets using the DAVID database (Sherman et al., 2022), and GO enrichment analyses included three aspects: Cellular Component (CC), Molecular Function (MF) and Biological Process (BP). The KEGG enrichment analysis can provide the pathways enriched by the overlapping targets. The above results were visualized using the bioinformatics platform (Tang et al., 2023).

### 2.4 Molecular docking

Based on topological analysis of Cytoscape 3.10.1 software (Shannon et al., 2003), the 2 proteins, top 2-degree value, were screened as target proteins for molecular docking. The three-dimensional crystal structure of 2 target proteins was gained from the RCSB-Protein Data Bank database (Berman et al., 2000). Simultaneously, the corresponding positive drug for the target proteins was retrieved from the Drugbank database.

The target proteins and ligands need to be pre-treated before molecular docking, including removing water molecules, protonate hydrogenation, and Gasteiger charges computation of target proteins and processing low-energy conformation of ligands. Molecular docking was then performed using Autodock 1.5.7 software using the blind docking method with default parameter settings, and the binding energy was used to evaluate the merit of the docking results.

## 3 Results

### 3.1 Fundamental features of apalutamide related AE reports, 2018–2024Q1

**FIGURE 2:**
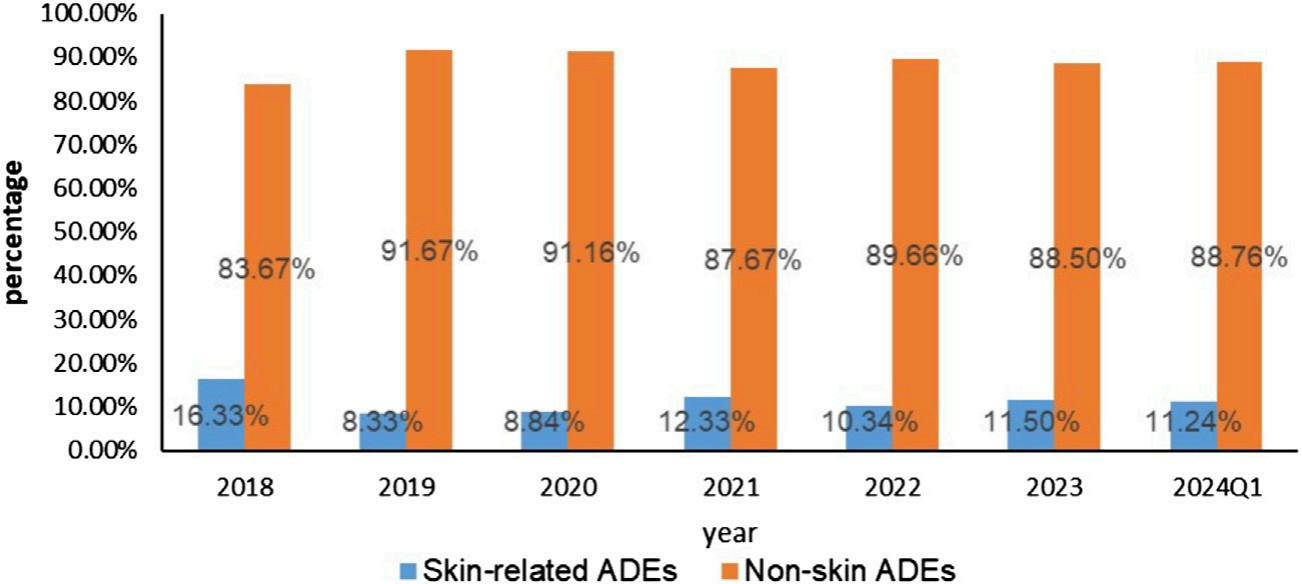
Skin adverse events and Non-skin adverse events reported of Apalutamide from Q1 2018 to Q1 2024.

Upon rigorous analysis of the FAERS database, a sum of 7,533 ADE reports came to the forefront wherein apalutamide was recognized as the primary suspect. Furthermore, 803 out of these accounts, which constitute 10.66% of the total, evidently pointed towards dermatotoxicity as an associated AE. Of all AE reports and skin-related AE reports, the U.S. had the highest number of reported cases, with consumers being the primary reporting population in total AE reports. However, the primary reporting population in skin-related AE reports was physicians. From the known age and weight data in the reports, 50.08% (604/1,206) of the reports were discovered to weigh less than 80 kg. Notably, 84.74% (3,482/4,109) of the reports included those older than 64, the chi-square test indicated that the low body mass group of <80 kg and the high age group of >64 years may be influential factors in the occurrence of skin AEs. Both the overall number of reported cases involving skin AEs and the total number of reported cases using apalutamide peaked in 2022. However, back in 2018, the ratio of reported cutaneous AEs incidents related to apalutamide reached its zenith, as shown in Table 3 and Figure 2.

**TABLE 3:** Basic information reported for relevant ADEs.

Categorization	Event	All ADE reports (n)	Skin-related ADEs (n)	*p-value*
Gender	Female	30	2	
	Male	6,879	657	
	Unknown	624	144	
Reporting States (top 5)	US	5,438	556	
	JP	608	86	
	CN	177	37	
	DE	171	11	
	FR	162	29	
Reporter	Consumer	2,777	177	
	Health-professional	1,388	177	
	Physician	1925	310	
	Other health-professional	288	38	
	Pharmacist	663	79	
	Unknown	492	22	
Age (year)	<65	627	30	*p <* 0.001
	≥65	3,482	333	
	Unknown	3,424	440	
Weight (kg)	<80	604	86	*p <* 0.001
	>80	602	39	
	Unknown	6,327	678	
Reporting year	2018	300	49	
	2019	732	61	
	2020	1,312	116	
	2021	933	115	
	2022	2,263	234	
	2023	1,548	178	
	2024Q1	445	50	

Unknown represents missing value.

### 3.2 AEs signal analysis

**FIGURE 3:**
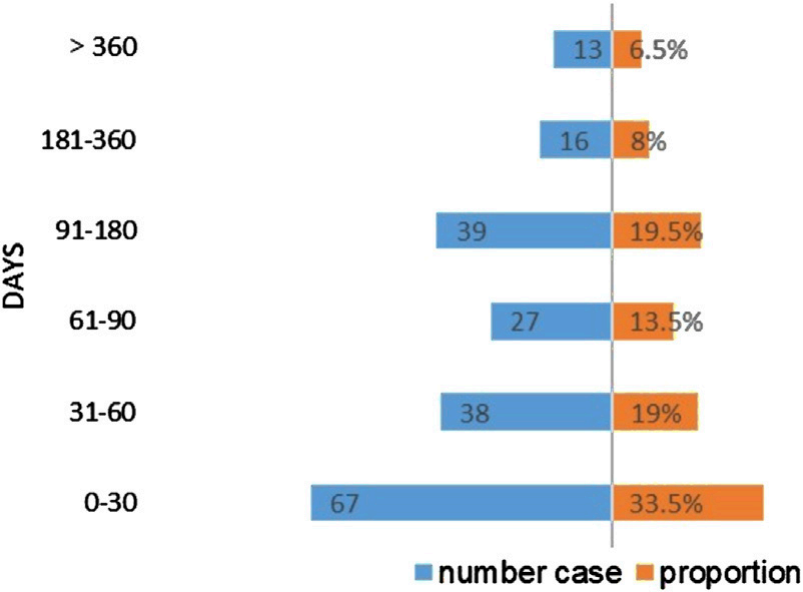
Time to onset of apalutamide-related skin AEs.

Following the signal detection utilizing the ROR and IC methodologies, we identified 21 PTs that surpassed the thresholds for both signals. The rash is the most common of the apalutamide skin AEs, with a notably high count of 769 reported cases, presenting a stark contrast to other adverse events. Serious skin adverse events such as Stevens-Johnson syndrome, toxic epidermal necrolysis and drug reaction with eosinophilia, and systemic symptoms have also been detected in drug reactions. Besides, a total of 10 signals were identified, which were not encompassed in clinical trials and drug instructions, including lichenoid keratosis, exfoliative dermatitis, and generalized exfoliative dermatitis. Exfoliative dermatitis and generalized exfoliative dermatitis exhibited the highest signal intensity based on the calculated ROR values (ROR = 46.84 and ROR = 24.05, respectively; Table 4). The time to AEs induction was characterized using quartiles, and an examination of 200 reports containing complete START_DT and EVENT_DT records showed a median onset time of 55 days for skin adverse events (interquartile range: 23.25–124.75), skin AEs occurring within 30 days are more frequently reported. Moreover, the Weibull distribution disclosed an early failure type with a Shape parameter <1, suggesting that skin AEs usually occur in the early stages of medication administration, as shown in Table 5 and Figure 3.

**TABLE 4:** Signal strength of apalutamide-associated skin PTs.

PT	Case number (n)	ROR (95%Cl)	IC (IC025)
Rash	769	7.92 (7.36–8.51)	2.91 (1.24)
Rash pruritic	47	4.17 (3.13–5.56)	2.06 (0.39)
Drug eruption	35	9.26 (6.64–12.91)	3.20 (1.53)
Rash erythematous	32	3.33 (2.35–4.71)	1.73 (0.07)
Drug reaction with eosinophilia and systemic symptoms	32	4.59 (3.24–6.49)	2.19 (0.53)
Dermatitis exfoliative generalized^a^	30	24.05 (16.77–34.48)	4.57 (2.90)
Rash maculo-papular	29	5.73 (3.98–8.25)	2.51 (0.85)
Stevens-Johnson syndrome	29	8.41 (5.83–12.11)	3.06 (1.40)
Toxic epidermal necrolysis	28	9.02 (6.22–13.09)	3.17 (1.50)
Dermatitis exfoliative^a^	16	46.84 (28.52–76.94)	5.51 (3.84)
Erythema multiforme	13	7.55 (4.38–13.02)	2.91 (1.24)
Dermatitis allergic^a^	12	4.26 (2.42–7.50)	2.09 (0.42)
Skin toxicity	12	9.06 (5.14–15.98)	3.17 (1.51)
Toxic skin eruption^a^	9	4.09 (2.13–7.87)	2.03 (0.36)
Lichenoid keratosis^a^	8	16.46 (8.21–33.02)	4.03 (2.36)
Rash vesicular	7	6.59 (3.14–13.84)	2.72 (1.05)
Exfoliative rash^a^	6	12.60 (5.65–28.13)	3.65 (1.98)
Onychomadesis^a^	3	3.97 (1.28–12.34)	1.99 (0.32)
Dandruff^a^	3	4.82 (1.55–14.96)	2.27 (0.60)
Dermatitis psoriasiform^a^	3	5.98 (1.92–18.57)	2.58 (0.91)
Miliaria^a^	3	6.82 (2.19–21.18)	2.76 (1.10)

^a^ Which one is not included in the instructions.

Exegesis: PT, preferred term, n, the number of reports collected on this PT; 95%Cl, 95% confidence interval of reporting odds ratio; IC025: the low 25% value proportional information components.

**TABLE 5:** Time to onset of Apalutamide-associated skin adverse events and Weibull distribution analysis.

	Time to onset (days)		Weibull distribution		
Drug	Case reports	Median (IQR)	Scale parameter:α (95% CI)	Shape parameter:β (95%CI)	Type
Apalutamide	200	55 (23.23–124.75)	102.29 (83.27–121.31)	0.80 (0.72–0.89)	Early failure

### 3.3 Network pharmacology results

**FIGURE 4:**
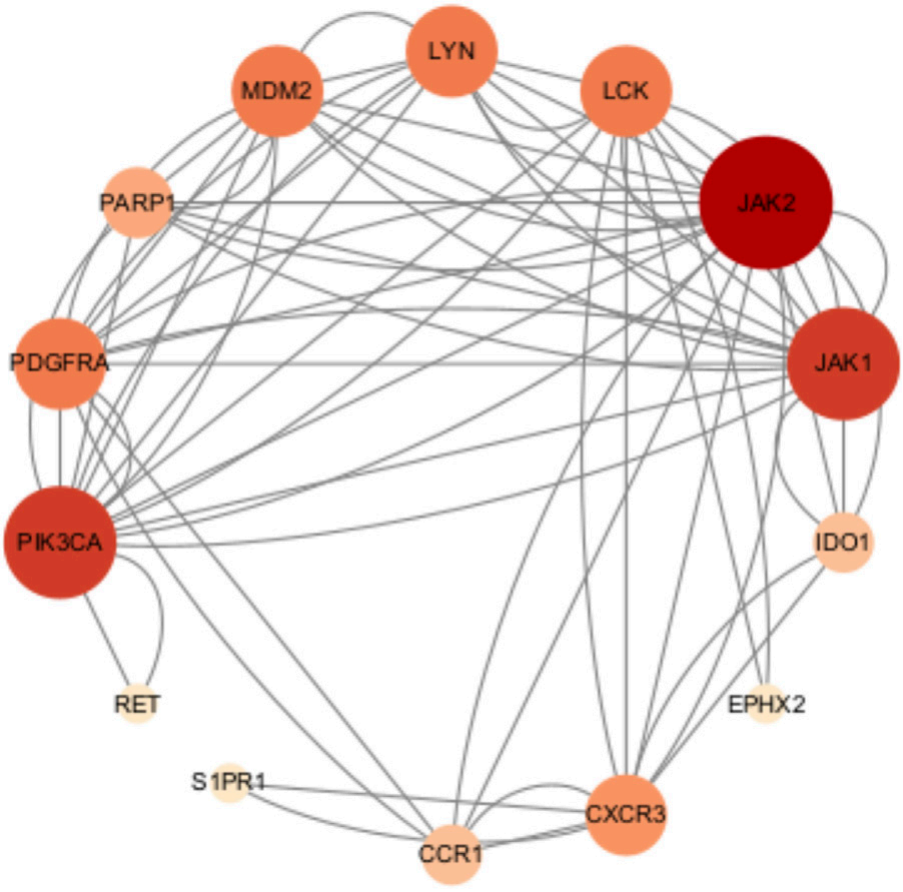
Protein-protein interaction diagram of apalutamide with SJS/TEN.

**FIGURE 5:**
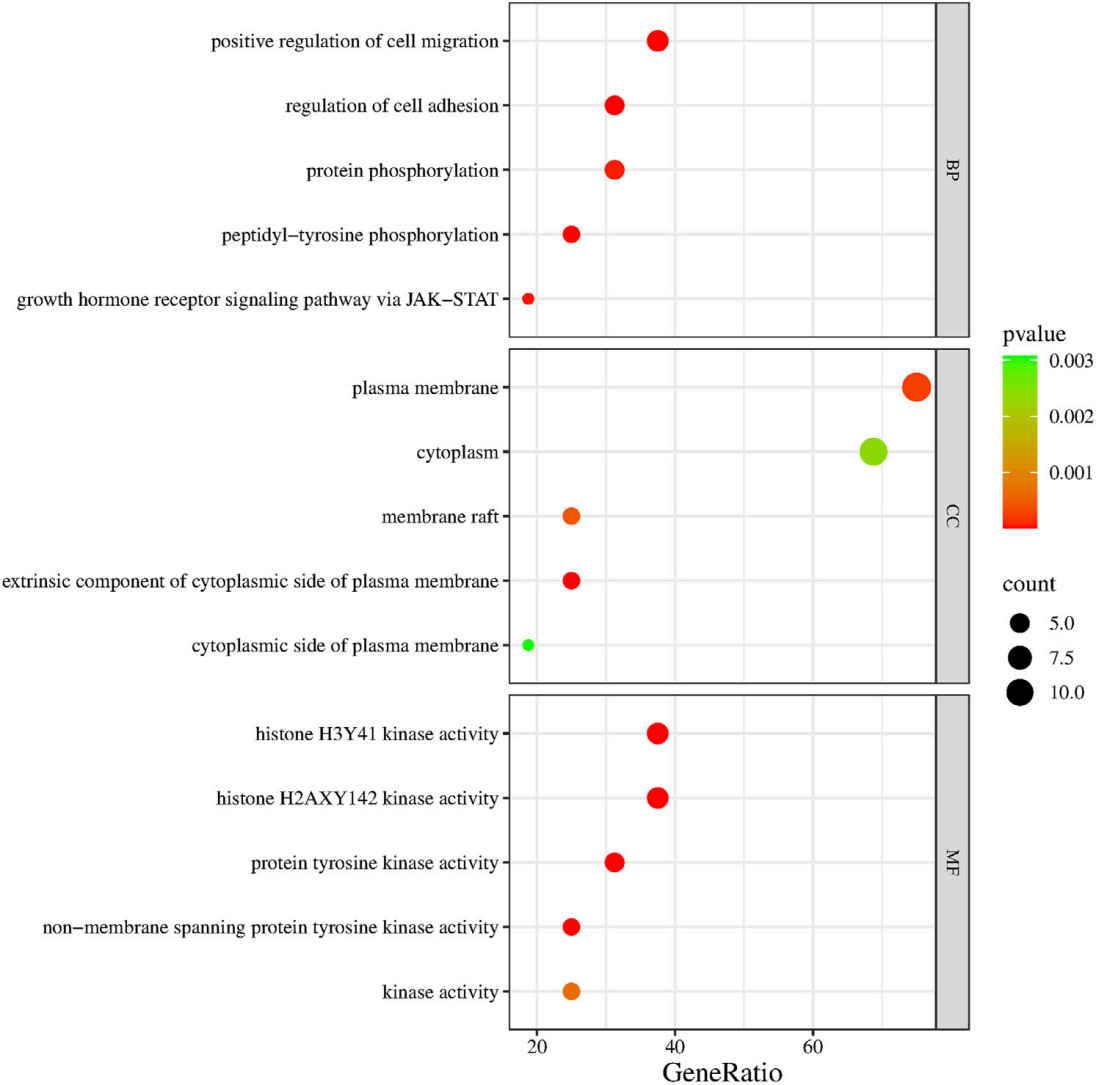
GO enrichment analysis bubble diagram.

**FIGURE 6:**
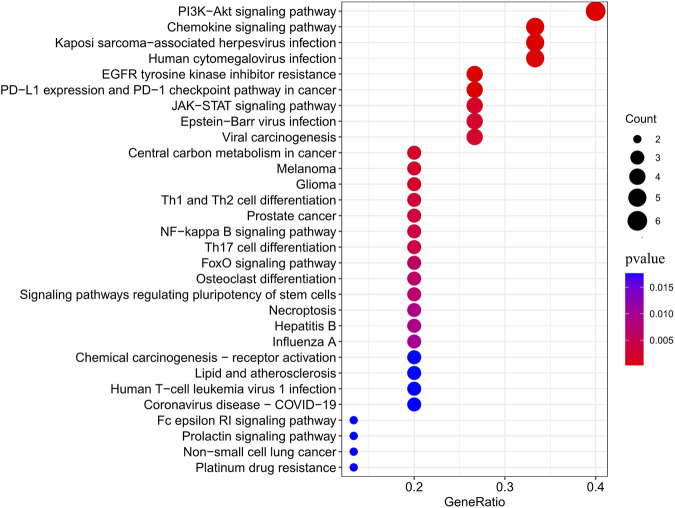
KEGG enrichment analysis bubble diagram.

As shown in Figure 4, JAK2, JAK1, PIK3CA, PDGFRA, LCK, MAM2, LYN, CXCR3, PARP1, CCR1, IDO1, SIPRI, EPHX2, and RET were common genes affected by apalutamide and SJS/TEN. Figure 5 shows the results of the first 15 GO enrichment analyses. The growth hormone receptor signaling pathway via JAK-STAT is one of the pathways of BP, which contains the target genes LYN, JAK2, and JAK1. The JAK-STAT signaling pathway is one of the pathways analyzed in the KEGG enrichment analysis (Figure 6), which contains the target genes PIK3CA, PDGFRA, JAK1, and JAK2.

### 3.4 Molecular docking results

**FIGURE 7:**
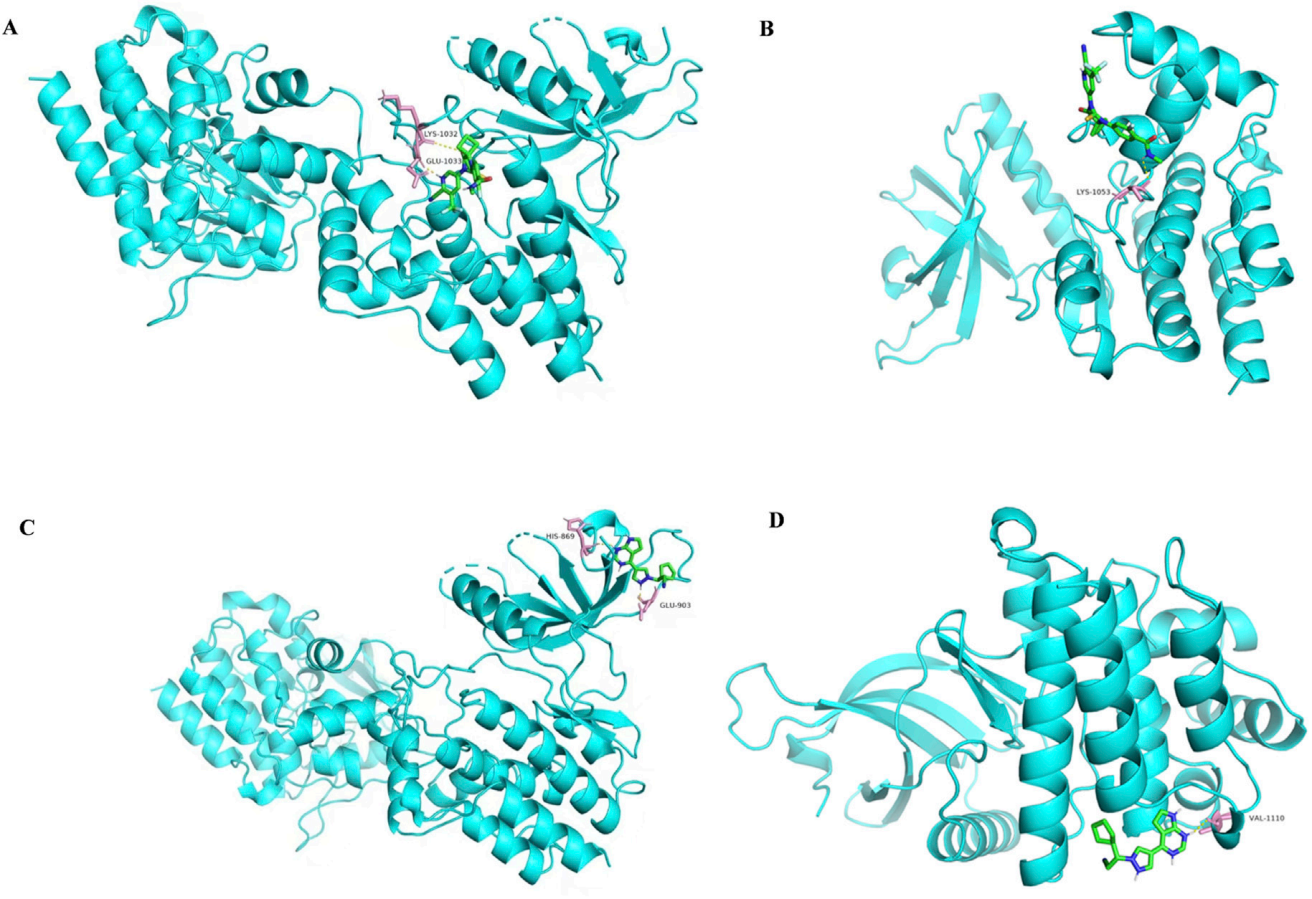
Molecular docking diagram of Apalutamide and Ruxolitinib with JAK1 and JAK2. **(A)** interaction of Aapalutamide with JAK1; **(B)** interaction of Aapalutamide with JAK2; **(C)** interaction of Ruxolitinib with JAK1; **(D)** interaction of Ruxolitinib with JAK2.

**FIGURE 8:**
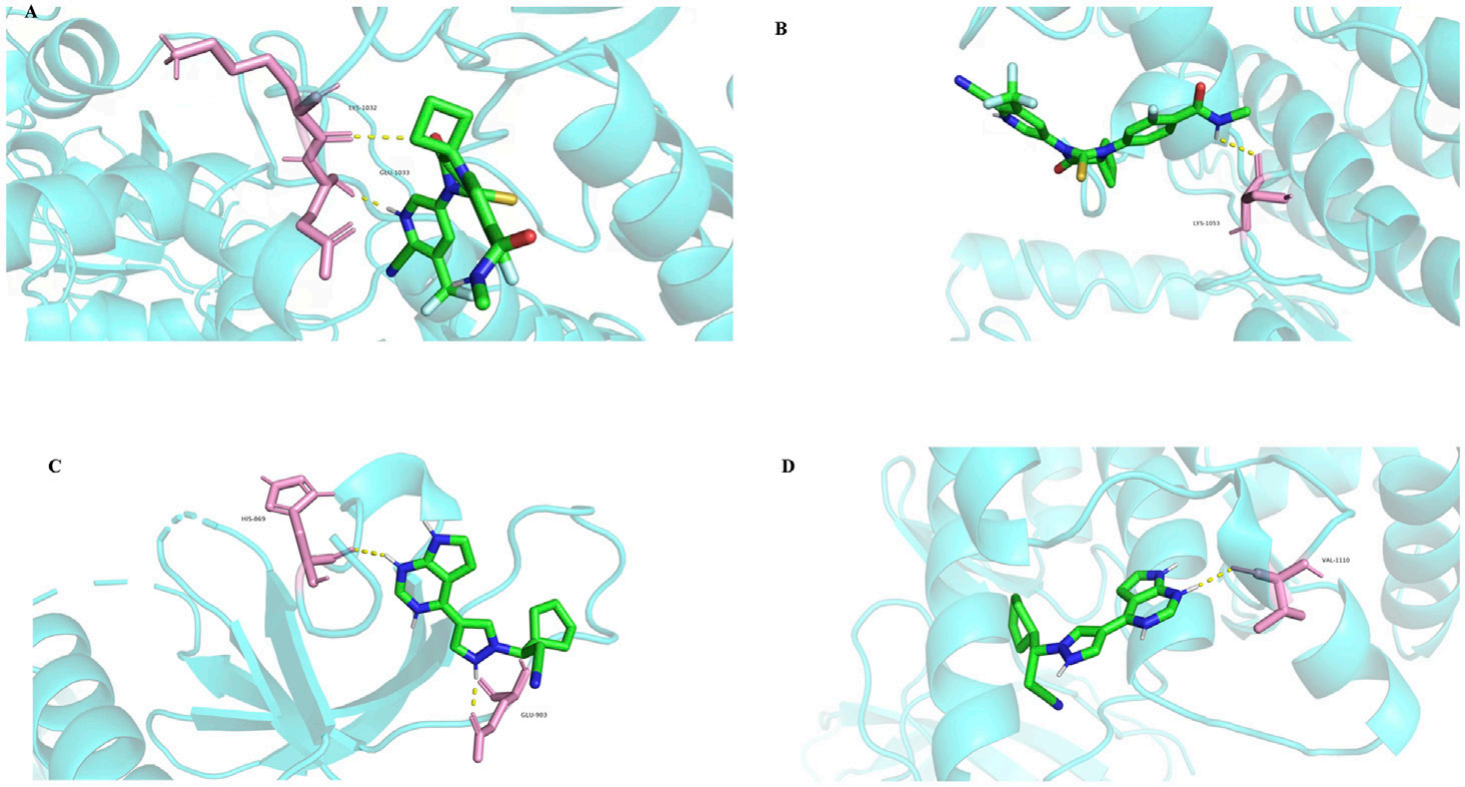
Molecular docking details diagram of Apalutamide and Ruxolitinib with JAK1 and JAK2. **(A)** interaction of Aapalutamide with JAK1; **(B)** interaction of Aapalutamide with JAK2; **(C)** interaction of Ruxolitinib with JAK1; **(D)** interaction of Ruxolitinib with JAK2.

Among the identified targets, JAK1 and JAK2 were the core targets in order of degree value. Ruxolitinib was selected as a positive drug for JAK1 and JAK2 target proteins because it is a potent, selective oral JAK1/JAK2 inhibitor whose binding energy to JAK1 and JAK2 is stable. The binding energies of apalutamide to the core targets JAK1 and JAK2 were −4.71 kJ/mol and −4.06 kJ/mol, respectively, which were smaller than the binding energies of Ruxolitinib to JAK1 and JAK2 (−4.71 kJ/mol < -4.01 kJ/mol, −4.06 kJ/mol < −3.87 kJ/mol, as shown in Figures 7, 8), indicating that the binding conformational stability of apalutamide with both JAK1 and JAK2 was stable.

## 4 Discussion

This study centered on skin AEs associated with apalutamide and revealed that physicians played a more valuable role in detecting skin AEs compared to other reporters. A study has displayed that low body weight may be a risk factor for the development of skin AEs with apalutamide (Katsuta et al., 2022). Patients <80 kg accounted for 68.8% (86/125) of the 125 reports of skin AEs with known body weight in this investigation, above the percentage of <80 kg in all reports (68.8% > 50.08%). In response to the epidemiology of prostate cancer, men over the age of 64 account for roughly 70% of cases (Zhai et al., 2020). In this study, reports from patients older than 64 made up over half of all reports; also, 91.74% (333/363) of patients older than 64 experienced skin AEs. Advanced age and low body weight may also be risk factors for apalutamide associated skin AEs in the current study, which is consistent with the findings of earlier associated research.

Among the 21 skin AEs, a few that were not anticipated attracted our attention and had not been reported in clinical trials of apalutamide. For example, mossy keratosis caused by apalutamide has been studied in only a few case reports, all of which considered it rare and serious (M.Tohyama, 2020; Shima et al., 2022; Cremante et al., 2023; Guglielmini et al., 2023). The disproportionality analyses plotted an intriguing correlation between apalutamide and desiccation, in comparison to other drugs within the FAERS database. However, nail changes were more frequently observed in prostate cancer patients receiving taxanes (Omlin et al., 2015; Dika et al., 2016). Changes in nails may also be influenced by factors besides apalutamide, there may also be an association with the disease itself, and long-term safety monitoring is necessary to confirm that.

Similar to the findings observed in SPARTAN and TITAN trials (Pan et al., 2022), rash exhibited a notably high frequency in this study, accompanied by evidence suggesting a correlation between the elevated incidence of apalutamide induced rash and the presence of 2-cyanopyridine in its chemical structure (Yano et al., 2024). TEN is a severe rash, which leads to mucous membrane erosions, extensive epidermal detachment, and severe systemic symptoms (Fouchard et al., 2000). TEN can easily be confused by the untrained eye with autoimmune blistering, erythema multiforme, acute generalized pustular pustulosis, staphylococcal scalded skin syndrome, and graft-versus-host disease (Kaffenberger and Rosenbach, 2014). Concerning the high death rate associated with TEN, early diagnosis and treatment are critical (Schwartz et al., 2013). A case report indicates that re-administration of apalutamide following the onset of TEN may be fatal (Wang et al., 2024), thus significantly limiting its use for subsequent treatment in patients. For that reason and to avoid the emergence of more serious outcomes, we recommend that physicians pay particular attention to skin AEs and that oncologists engage with dermatologists once skin AEs occur to enable them to promptly implement specialized therapy.

Corticosteroids, cyclosporine, plasmapheresis, and immunoglobulins have been utilized in the treatment of SJS/TEN; however, evidence suggests no striking difference in their efficacy (Ogiji et al., 2024). Network pharmacological results presented that SJS/TEN may be caused by the binding of apalutamide to the proteins JAK1 and JAK2. There are 4 members in the Janus kinase (JAK) family: JAK1, JAK2, JAK3, and TYK2. JAK mediates the signaling pathways for cytokines and growth factors which are involved in inflammation, hematopoiesis, and immune responses (Drożdżal et al., 2021). It has been proven that increased IL-15 levels are linked to the development and death of SJS/TEN, and the downstream signaling of IL-15 is mediated by JAK signal transduction and activator of transcription (JAK-STAT) (Stern and Divito, 2017; Su et al., 2017). Nordmann et al. utilized deep visual proteomics to discover that significant upregulation of the JAK/STAT pathway is a key pathogenic driver of TEN (Nordmann et al., 2024).

JAK inhibitors partially inhibit the activity of JAK enzymes and regulate intracellular signaling, consequently reducing the phosphorylation and activation of STAT proteins (Drożdżal et al., 2021), reducing the expression of IL-15, therefore JAK inhibitors have also been considered as a potential treatment for SJS/TEN (Xia et al., 2023). Nordmann et al. also observed an improvement in symptoms in TEN model mice following JAK inhibitors treatment, and seven patients with TEN or SJS-TEN overlap syndrome, including those refractory to high-dose systemic corticosteroid therapy, were discharged from hospital after successful recovery following treatment with a JAK inhibitor (Nordmann et al., 2024). JAK inhibitors have verified promising efficacy in inflammatory skin diseases such as lichen planus, dermatomyositis, and atopic dermatitis (Huang and Armstrong, 2023; Yuan et al., 2024). Besides, evaluation of the efficacy of JAK inhibitors for the treatment of SJS/TEN is currently underway (NCT06474078, NCT06119490).

Meanwhile, related work has established that in some metastatic castration resistant prostate cancer patients, long-term AR inhibitors treatment creates a selective pressure to generate AR-independent tumors with an altered profile. The altered spectrum is associated with activation of the JAK/STAT pathway, which drives the transformation of tumor cells from an epithelial state to a mesenchymal state (EMT), a transformation that renders the tumor cells unresponsive to androgen signaling and resistant to AR inhibitors. In the presence of high activation of the JAK-STAT pathway, the combination of a JAK inhibitor with an FGFR inhibitor enables tumor cells to switch from a drug-resistant phenotype (e.g., EMT) back to the luminal state with high AR expression, thereby restoring sensitivity to androgen receptor inhibitors (Chan et al., 2022; Deng et al., 2022). Considering the enhanced effects of JAK inhibitors on skin inflammatory diseases and their potential to overcome resistance to androgen receptor-targeted therapy in prostate cancer, we hypothesize that the combination of JAK inhibitors and apalutamide may hold promise for prostate cancer treatment, but at present there are no clinical studies of this combination therapy, and it may be a logical direction to explore in the future.

Through the use of the FAERS database and Network pharmacology, this paper contributes to our understanding of apalutamide skin AEs; however, it is important to acknowledge that this study has some limitations. As a self-reporting database, FAERS is unable to determine the precise number of individuals taking medication or the details of adverse events that occurred during a specific time frame, making it impossible to determine the incidence of a particular AE. We still need laboratory studies based on molecular docking data to confirm the link between apalutamide and JAK1/2.

## 5 Conclusion

The current study inspected apalutamide induced skin adverse events and discovered that low body weight and advanced age may be risk factors for the development of skin adverse events. The median time to the occurrence of all skin adverse events was 55 days. When compared to other medications, there is a notable association between apalutamide and SJS/TEN, this association may be caused by apalutamide’s binding to JAK1/JAK2. Future research is required to confirm the connection between skin adverse events and Janus kinase and apalutamide.

## Data Availability

The datasets presented in this study can be found in online repositories. The names of the repository/repositories and accession number(s) can be found in the article/supplementary material.
